# Depression among health workers caring for patients with COVID-19 in Egypt

**DOI:** 10.1186/s41983-021-00394-1

**Published:** 2021-10-18

**Authors:** Hayam Mohamed Elgohary, Mohammad Gamal Sehlo, Medhat Mohamed Bassiony, Usama Mahmoud Youssef, Dina Sameh Elrafey, Shimaa Ibrahim Amin

**Affiliations:** 1 Department of Psychiatry, Faculty of Medicine, Zagazig University, PO Box 44519, Zagazig, Egypt; 2 Department of Community, Environmental and Occupational Medicine, Faculty of Medicine, Zagazig University, Zagazig, Egypt

**Keywords:** Major depression disorder, Healthcare workers, COVID-19 pandemic, Egypt

## Abstract

**Background:**

Health care workers caring for patients with COVID-19 pandemic are prone to extraordinary stressors and psychological problems. The aim of this study was to estimate the prevalence and risk factors of major depressive disorder among health care providers who are caring for patients with COVID-19.

**Methods:**

Two hundred-seventy of health care workers were screened for depressive symptoms by DASS-21 Questionnaire. Only 152 of the participants accepted to be interviewed using SCID-I for diagnosis of major depressive disorder.

**Results:**

According to DASS-21, 28.1% of HCWs had mild-to-moderate depressive symptoms, and 64.8% with severe symptoms. Of 152 who were interviewed using SCID-I, 74.3% were diagnosed with major depression disorder.

Young age, decreased sleep hours, female sex, past history of a psychiatric disease, fear of COVID-19 infection for themselves or their relatives, and fear of death with COVID-19 for themselves or their relatives were significant predictors for major depressive disorder and its severity.

**Conclusion:**

Major depressive disorder is common among HCWs during COVID-19 pandemic. Screening for depression, particularly for young females, and early treatment are recommended.

## Background

Lower respiratory tract infections are the communicable diseases with the high mortality rate around the world [1]. In December 2019, a highly infectious acute respiratory syndrome caused by a novel coronavirus (SARS-CoV-2) emerged in Wuhan, China. On March 11th 2020, the World Health Organization (WHO) declared COVID-19 a pandemic [2].

According to previous studies from SARS or Ebola epidemics, the onset of a sudden and immediately life-threatening illness may lead to extraordinary stressors on health care workers (HCWs) [3]. Increased workload, physical exhaustion, insufficient personal equipment, fear of infection, and the need to make ethically difficult decisions may have dramatic effects on their physical and mental well-being. Isolation, loss of social support and fear of transmission of infection to relatives and friends could make HCWs liable to mental health problems, such as fear, anxiety, insomnia and depression [4, 5]. Now in COVID-19 era, health care workers are first-line fighters treating patients with COVID-19 and they are exposed to long and distressing work shifts to meet health requirements which may exceed their individual coping skills [6].

Previous studies have reported the prevalence and factors associated with psychological outcomes in HCWs during past infectious disease outbreaks [7–9]. However, the impact of the current COVID-19 pandemic on the psychological well-being of medical staff is yet needed to be studied.

There are only few studies that evaluated depression and its risk factors among HCWs worldwide [10], but they used self-rating scales for assessment of depressive symptoms. The aim of the present research is to estimate the prevalence and potential risk factors contributing to major depressive disorder, which diagnosed by structural interview using SCID-I, among Egyptian health care workers who are caring for COVID-19 patients.

## Methods

The data were collected during the period of March–September, 2020. The participants were the HCWs (doctors, nurses, pharmacists, technicians and paramedical workers) who were working in the isolation units for COVID-19 patients, at a university hospital in Egypt. Participants were selected using a convenience sampling technique. Inclusion criteria: age 18–60 years, both sexes and who accepted to participate. The sample size was calculated according the Epi Info 6.0, at 80% power of the study, 95% confidence level [11], with minimum sample size 260. We recruited 300 participants to overcome dropout.

The first step: An electronic Google form survey was sent to the participants via their E-mails (*N* = 300). It was designed by the authors to collect: 1—socio-demographic data (age, gender, marital status, residence, level of education, occupation and telephone number) and other clinical data; 2—data related to Depression, Anxiety and Stress Scale (DASS-21) for assessment of depressive symptoms. Prior to conducting the survey, the purpose of the study was explained to participants. Only 270 complete the survey and 30 gave incomplete response, so they were excluded.

The second step: All participants who complete the depression subscale of DASS-21 (*N* = 270) were invited for further evaluation by applying Structured Clinical Interview for DSM-5 disorders (SCID interview) for diagnosis of Major depressive disorder (MDD). The interviews were done in isolation units considering infection control by using the personal protective equipment PPE. Only 152 completed the SCID-I evaluation interview.

### Measures

A. The Depressive subscale of Depression anxiety stress scale (DASS-21): The Depression Anxiety Stress Scale (DASS-21) is a self-report tool containing 21 items that assess depression, anxiety, and stress. There are subscales with 7 items each (depression, anxiety and stress). Each item is scored on a 4-point Likert scale (0 = did not apply to me at all and 3 = applied to me very much, or most of the time), with a higher score indicating more severe levels of distress. The summation of each scale, then multiplied by two to convert to full scale scores. Each score ranged from 0 to 42. Participants with cutoff scores of ≥ 10 for the depression dimension (≥ 10 as “mild depression”, ≥ 14 as “moderate”, ≥ 21 as “severe”, and ≥ 28 as “extremely severe”), ≥ 8 in anxiety (≥ 8 as “mild anxiety”, ≥ 10 as “moderate”, ≥ 15 as “severe”, and ≥ 20 as “extremely severe”), and ≥ 15 in stress (≥ 15 as “mild anxiety”, ≥ 19 as “moderate”, ≥ 26 as “severe”, and ≥ 34 as “extremely severe”) were considered to have these disorders. The scale has been shown to have good test–retest reliability, internal consistency and convergent validity [12].
B. Structured Clinical Interview for DSM-5 disorders, clinician version (SCID-5**-**CV). For diagnosis of Major depressive disorder [13]

Data analysis was performed using the statistical package for social sciences software (SPSS version 20). The qualitative data were presented in the form of number and percentage. The quantitative data were presented in the form of mean and standard deviation. Groups were compared using independent sample *t* test for quantitative parameters. For qualitative variables, chi-square was used as a test of significance of differences among groups. Linear and logistic regression was used in the analysis of the predictors for depression. A *P*-value < 0.05 was considered to indicate statistical significance.

## Results

This study included 270 health care workers (HCWs). The studied group age ranged from 18 to 52 years with mean 34.98 ± 6.27 years. 57% of HCWs were female. More than 72% of them had post-graduate education. Most frequent occupations among the participants were physicians and nurses (70.7% and 16.3%, respectively). The most common specialties among physicians were clinical pathology, anesthesia, ICU and pulmonary (10.4%, 10% and 7%, respectively). Most of the HCWs were married (79.6%), from urban area (77.4%) and non-smokers (84.4%). Only 9.6% of the studied HCWs had past history of psychiatric disorders and 12.6% had family history of psychiatric disorders.

**Fig. 1:**
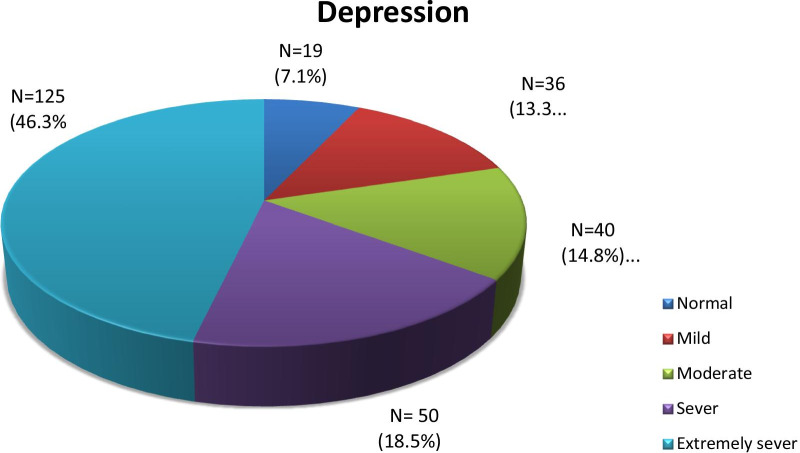
Frequency of depressive symptoms according to DASS-21 questionnaire among the HCWs

Among HCWs, 2.2% reported suicidal thoughts. The working hours ranged from 4 to 14 with mean 9.31 ± 3.1 while sleeping hours ranged from 3 to 10 with mean 6.57 ± 1.27. DASS-21 was answered by 270 of the participants (response rate: 90%). HCWs without depressive symptoms were 7% (*N* = 19), mild 13.3 (*N* = 36), moderate 14.8% (*N* = 40), severe 18.5% (*N* = 50) and extremely severe depressed HCWs represented 46.3% (*N* = 125) as shown in Fig. 1.

Table 1 shows the comparison between HCWs with severe-to-extremely severe depression and those with mild-to-moderate depression. There was a statistical significant decrease in mean age and sleep hours among HCWs with severe and extremely severe depression. In addition, there was a statistical significant increase in frequency of severe and extremely severe depression among females, HCWs who had past history of psychiatric disorder, fear of working during COVID-19 pandemic, fear of being infected with COVID-19, fear of infection of a family member with COVID-19, fear of death with COVID-19 and fear of a family member death with COVID-19. The same factors remain significant after linear regression analysis for predictors of depression severity according to DASS-21 score as shown in Table 2.

**Table 1:** Comparison between those who are normal, mild and moderate depression and those with severe and extremely severe depression according to DASS-21 depression score

Variable	Normal mild and moderate (*N* = 95)	Severe and extremely severe (*N* = 175)	Test	
Age				
Mean ± SD	36.41 ± 6.59	34.20 ± 5.97		
Range	24–50	18–52	**2.80**	0.005*
Working hours				
Mean ± SD	8.73 ± 2.97	9.63 ± 3.15		0.78
Range	4–14	4–14	0.28	NS
Sleep hours				
Mean ± SD	6.78 ± 0.95	5.54 ± 1.69		
Range	6–10	3–10	**6.60**	< 0.001**

Variable	*N*	*N*	%	*N*	%	*χ*^2^	*P*
Sex							
Female	154	44	28.6	110	71.4	**6.88**	0.009*
Male	116	51	44	65	56		
Education							
Intermediate education	25	9	36	16	64	0.67	0.72
High education	50	20	40	30	60		NS
Postgraduate	195	66	33.8	129	66.2		
Occupation							
Doctors	191	71	37.2	120	62.8	0.26	0.60
Nurses	44	18	40.9	26	59.1		
Pharmacists	19	11	57.9	8	42.1		
Technicians	12	3	25	9	75		NS
Paramedical workers	4	0	0	4	100		
Marital status							
Single	39	16	41	23	59	0.19	0.60
Married	215	81	37.6	134	62.4		
Divorced	15	6	40	9	60		NS
Widow	1	0	0	1	100		
Residence							
Urban	209	71	34	138	66	0.60	0.44 NS
Rural	61	24	39.3	37	60.7		
Smoking							
No	228	85	37.3	143	62.7	2.82	0.09
Yes	42	10	23.8	32	76.2		NS
Past history of psychiatric disorders							
No	244	93	38.1	151	61.9	**9.54**	0.002**
Yes	26	2	7.7	24	92.3		
Family history of psychiatric disorders							
No	236	84	35.6	152	64.4	0.14	0.71
Yes	34	11	32.4	23	67.6		NS
Fear of working during COVID-19							
No	72	33	45.8	39	54.2	**4.88**	0.02*
Yes	198	62	31.3	136	68.7		
Fear of infection of COVID-19							
No	52	25	48.1	27	51.9	**4.69**	0.03*
Yes	218	70	32.1	148	67.9		
Fear of transmission of infection to relatives							
No	10	6	60	4	40	**8.05**	**0.004**
Yes	260	56	21.6	204	78.4		
Had infected with COVID-19							
No	253	93	36.7	160	63.3	0.13	0.71
Yes	17	7	41.2	10	58.8		NS
Had family member infected with COVID-19							
No	216	100	46.2	116	53.8	0.13	0.71
Yes	54	24	18.5	30	81.5		NS
Fear of death with COVID-19							
No	96	42	43.8	54	56.2	**4.79**	0.03*
Yes	174	53	30.5	121	69.5		
Fear of family member death with COVID-19							
No	19	12	63.2	7	36.8	**7.70**	**0.005**
Yes	251	80	35.1	171	64.9		
Suicidal thoughts							
No	264	98	37.1	166	62.9	3.50	0.06
							NS
Yes	6	0	0	6	100		

Bold values highlight the significant results

*SD* standard deviation, *Test* independent t test, *χ*^2^ chi-square test

*NS* non-significant (*P* > 0.05)

*Significant (*P* < 0.05); **Highly significant (*P* < 0.001)

**Table 2:** Linear regression analysis for predictors for severity of depression according to DASS-21 depression score among the studied group

	Unstandardized coefficients		Standardized coefficients	*t*	*P*	95.0% CI	
	*B*	SE	Beta				
Age	**− 0.38**	**0.09**	**− 0.24**	**− 4.23**	< 0.001**	**− 0.56**	**− 0.20**
Female sex	**2.15**	**0.07**	**0.21**	**3.70**	0.03*	**0.15**	**4.65**
Education	− 1.25	1.10	− 0.08	− 1.14	0.26 NS	− 3.42	0.91
Occupation	− 1.38	0.56	− 0.15	− 2.44	0.22 NS	− 2.49	0.27
Marital status	0.42	1.01	0.02	0.42	0.68 NS	− 1.57	2.40
Residence	0.35	1.43	0.01	0.24	0.81 NS	− 2.48	3.17
Past history of psychiatric disorder	**8.86**	**1.85**	**0.26**	**4.79**	< 0.001**	**5.22**	**12.51**
Family history of psychiatric disorders	− 0.88	1.67	− 0.03	− 0.53	0.60 NS	− 4.16	2.41
Smoking	0.73	1.73	0.07	0.42	0.67 NS	− 2.67	4.13
Fear of working during COVID-19	1.59	1.37	0.19	1.16	0.25 NS	1.15	4.28
Fear of infection of COVID-19	**4.52**	**1.67**	**0.18**	**2.72**	0.01*	**1.24**	**7.80**
Fear of transmission of infection to relatives	**13.07**	**3.76**	**0.31**	**3.48**	0.001*	**5.66**	**20.48**
Had infected with COVID-19	0.10	1.22	0.01	0.08	0.94 NS	− 2.31	2.51
Had family member infected with COVID-19	0.08	1.81	0.00	0.04	0.97 NS	− 3.48	3.65
Fear of death with COVID-19	**4.74**	**2.16**	**0.12**	**2.18**	0.03*	**9.02**	**0.46**
Fear of family member death of COVID-19	**4.36**	**1.41**	**0.17**	**3.09**	0.002*	**1.58**	**7.15**
Suicidal thoughts	0.37	1.40	0.07	0.28	0.83 NS	− 2.44	3.26
Working hours	0.02	0.13	0.01	0.16	0.87 NS	− 0.24	0.28
Sleep hours	**− 1.10**	**0.38**	**− 0.16**	**− 2.86**	0.005*	**− 1.85**	**− 0.34**

Bold values highlight the significant results

*NS* non-significant (*P* > 0.05)

*Significant (*P* < 0.05); **Highly significant (*P* < 0.001)

**Fig. 2:**
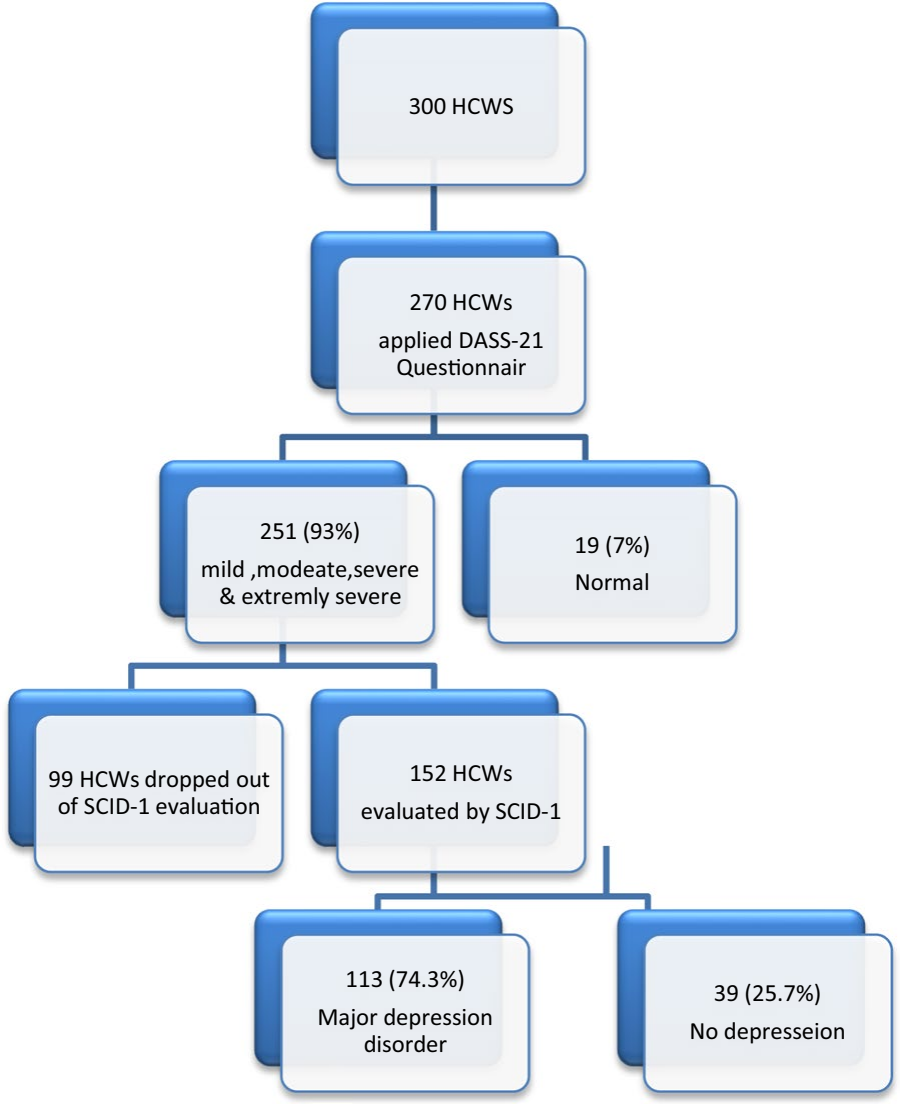
Diagram for the whole sample

SCID-I evaluation was conducted on 152 HCWs who reported depression by DASS-21 scale and the evaluation revealed that 113 of them (74.3%) diagnosed with major depression (Fig. 2).

Depressed HCWs had statistically decreased mean age and decreased sleep hours than non-depressed. Moreover, frequency of depression was statistically increased among females, those who had a past history of psychiatric disorders, presence of fear of being infected with COVID-19, fear of infection of a family member with COVID-19, fear of death with COVID-19 and fear of a family member death with COVID-19 as shown in Table 3. The same factors remain significant after applying binary logistic regression analysis as shown in Table 4.

**Table 3:** Comparison between those who are not depressed versus those who are depressed according to SCID-1 evaluation

Variable	No major depression disorder (*N* = 39)	Major depression disorder (*N* = 113)	Test	
Age				
Mean ± SD	36 ± 6.74	33.75 ± 5.65	**2.04**	0.04*
Median (range)	36 (23–52)	34 (18–46)		
Working hours				
Mean ± SD	8.38 ± 2.62	10.23 ± 5.25	1.59	0.11
Median (range)	8 (5–12)	8 (4–24)		NS
Sleep hours				
Mean ± SD	6.62 ± 1.16	5.40 ± 1.83	**3.90**	< 0.001*
Median (range)	7 (3–8)	5 (3–15)		

Variable		*N*	*N*	%	*N*	%	*χ*^2^	*P*
Sex								
Female		93	17	18.3	76	81.7	**6.84**	0.009**
Male		59	22	37.3	37	62.7		
Education								
Intermediate education		16	3	18.8	13	81.3	0.93	0.63
High education		24	5	20.8	19	79.2		NS
Postgraduate		112	31	27.7	81	72.3		
Occupation								
Doctors		109	31	28.4	78	71.6		
Nurses		24	5	20.8	19	79.2		
Pharmacists		8	2	25	6	75	2.43	0.66
Technicians		9	1	11.1	8	88.9		NS
Paramedical workers		2	0	0	2	100		
Marital status								
Single		17	3	17.6	14	82.4		
Married		121	33	27.3	88	72.7		
Divorced		13	3	23.1	10	76.9	1.13	0.77
Widow		1	0	0	1	100		NS
Residence								
Urban		123	34	27.6	89	72.4	1.33	0.25
Rural		29	5	17.2	24	82.8		NS
Smoking								
No		124	34	27.4	90	72.6	1.10	0.30
Yes		28	5	17.9	23	82.1		NS
Past history of psychiatric disorders								
No		130	39	30	91	70	**8.88**	0.003*
Yes		22	0	0	22	100		
Family history of psychiatric disorder								
No		132	34	25.8	98	74.2	0.01	0.94
Yes		20	5	25	15	75		NS
Fear of working during COVID-19								
No		38	13	34.2	25	65.8	1.94	0.16
Yes		114	26	22.8	88	77.2		NS
Fear of infection of COVID-19								
No		28	12	42.9	16	57.1	**5.32**	0.02*
Yes		124	27	21.8	97	78.2		
Fear of transmission of infection to relatives								
No		5	4	80	1	20	**8.00**	0.005*
Yes		147	35	23.8	112	76.2		
Had infected with COVID-19								
No		129	36	27.9	93	72.1	2.26	0.13
Yes		23	3	13	20	87		NS
Had family member infected with COVID-19								
No		12	1	8.3	11	91.7	2.05	0.15
Yes		140	38	27.1	102	72.9		NS
Fear of death with COVID-19								
No		108	33	30.6	75	69.46	**3.94**	0.03*
Yes		44	6	13.6	38	86.4		
Fear of death of family member with COVID-19								
No		47	17	36.2	30	63.8	**4.69**	0.04*
Yes		105	22	21	83	79		
Suicidal thoughts								
No		149	47	31.5	102	68.5	1.37	0.24
Yes		3	0	0	3	100		NS

Bold values highlight the significant results

*SD* standard deviation, *Test* independent t test, *χ*^2^ chi-square test, *NS* non-significant (*P* > 0.05)

*Significant (*P* < 0.05); **Highly significant (*P* < 0.001)

**Table 4:** Binary logistic regression analysis for predictors of major depression disorder according to SCID-1 among the studied group

	*B*	S.E	Wald	*P*	OR	95% CI	
Young age	**0.24**	**0.08**	**8.04**	0.005*	**1.27**	**1.08**	**4.49**
Female sex	**2.36**	**0.90**	**6.88**	0.009*	**6.25**	**3.13**	**18.36**
Education	1.16	1.59	0.54	0.47 NS	3.19	0.14	71.80
Occupation	− 0.53	2.87	0.03	0.85 NS	0.59	0.002	164.62
Marital status	0.32	2.23	0.02	0.89 NS	1.38	0.02	108.49
Past history of a psychiatric disorder	**6.06**	**2.43**	**6.20**	0.01*	**7.63**	**3.64**	**9.62**
Residency	2.06	2.66	2.30	0.91 NS	1.19	0.04	32.33
Family history of psychiatric disorder	− 0.63	1.00	0.39	0.53 NS	0.53	0.08	3.80
Smoking	1.53	1.06	2.09	0.15 NS	4.63	0.58	36.97
Fear of working with during COVID-19	0.50	0.80	0.39	0.53 NS	1.65	0.35	7.85
Fear of infection with COVID-19	**2.25**	**1.10**	**4.17**	0.04*	**3.95**	**1.09**	**5.88**
Fear of transmission of COVID-19 to a family member	**2.83**	**1.32**	**4.65**	**0.02**	**4.11**	**1.23**	**6.71**
Had infected of COVID-19	1.38	1.11	1.56	0.21 NS	3.99	0.45	35.19
Had a family member infected with COVID-19	0.44	0.81	0.30	0.59 NS	1.55	0.32	7.58
Fear of death with COVID-19	**1.85**	**1.02**	**3.26**	0.03*	**2.16**	**1.04**	**5.17**
Fear of family member death of COVID-19	**1.79**	**1.01**	**3.09**	**0.04**	**2.01**	**1.02**	**4.93**
Suicidal thoughts	1.13	1.20	2.50	0.70 NS	2.16	0.73	8.12
Working hours	0.12	0.07	2.82	0.09 NS	1.13	0.98	1.31
Sleep hours	**0.69**	**0.29**	**5.62**	0.018*	**2.00**	**1.13**	**3.55**

Bold values highlight the significant results

*NS* non-significant (*P* > 0.05)

*Significant (*P* < 0.05); **Highly significant (*P* < 0.001)

## Discussion

To our knowledge, this is the first research to use the structured clinical interview for DSM (SCID-I) to detect major depression disorder (MDD) among health care workers (HCWs) who were treating patients with COVID-19 in Egypt.

The current study was a cross-sectional survey that enrolled 270 HCWs and revealed a high prevalence of depressive disorder. Overall respondents, 251(93%) had depressive symptoms based on DASS-21. Twenty-eight percent of HCWs had mild-to-moderate depressive symptoms, and 65% with severe or extremely severe depressive symptoms. By applying SCID-I, 74.3% of HCWs were diagnosed with MDD. In a previous study during the acute SARS outbreak, 89% of HCWs who were in high-risk situations reported psychological symptoms [14]. An Egyptian study similarly reported a high prevalence of severe-to-extremely severe depressive symptoms among Egyptian physicians during the COVID-19 pandemic, the majority (63%) suffered from severe or extremely severe depressive symptoms [15]. Also, the prevalence of depressive symptoms among health care providers was (78.1%) in Jordan [16].

Two Egyptian studies and another Saudi one reported that severe depression represents 20.5%, 14%, 5.8%, consequently these results are considered low in comparison to the current one. This can be explained by the using of different measures noting the in this study the authors used the confirmatory diagnostic clinical interview according to the DSM [17–19].

Another Turkish study reported that 64.7% of physicians had symptoms of depression [20]. Many studies were performed in China: two of them reported that the prevalence of depressive symptoms among health care providers was 50.4% [21] and 56.0% [22].

The current results reported higher prevalence of depressive symptoms among HCWs than that of other studies, most probably due to the different conditions in which the HCWs are working in Egypt. HCWs did not deal with such a catastrophic and emerging pandemic before. Moreover, they are facing highly infectious disease with uncertain outcome with deficient infection control supplements and shortage of protective equipment that cause overburden and extraordinary stressors over them [22–25]. In addition, there are other factors that may explain the high prevalence of depression among HCWs generally such as workloads, burnout, insufficient time to take care of their families during the pandemic, social stigma, health anxiety and fear towards COVID-19 infection and reluctance of the society to support them [26–30].

The current study found that young age was associated with higher scores of depression among HCWs. This finding is supported by a recent Jordanian study conducted during the COVID-19 pandemic, which demonstrated that the young age group of HCWs had a significantly high risk to develop depression [16]. A study in Saudi Arabia stated that the age group from 30 to 39 had a slightly high level of depression and anxiety [18]. This is also consistent with recent studies that demonstrate an elevated incidence of psychiatric disorders in younger adults [31, 32]. This could be attributed to the less adaptive manner of responding to stressors that may justify this result [33] and the age-related bio-psychosocial changes [31]. Moreover, young HCWs had to spend a long time in emergency units in close contact with COVID-19 patients to gain clinical experiences, causing a high level of stress and fear of acquiring the infection.

The results showed that fear of being infected with COVID-19 or transmitting the infection to family members, fear of death, or family members’ death with COVID-19, were associated with an increase in the severity of depression among HCWs. In line with these findings, recent studies in China and Nepal reported that medical staffs were fearful about transmitting the virus to their families [21, 23, 28]. A recent study reported that the most concerns regarding the COVID-19-related fears among medical staff in Egypt were the fear of being infected and the fear of transmission of the disease to their families [34]. HCWs may isolate themselves to lower the risk of infecting their family members. Thus the absence of emotional support could attribute the increased psychological distress and affect HCWs mental well-being [35].

The current study found that female HCWs were six times more likely to have depression than male HCWs. Similarly, the results of the Saudi, Jordanian and Egyptian studies indicate that being female increased the risk of depression among health care providers during the COVID-19 pandemic [16, 17, 36, 37]. HCWs with past history of psychiatric illness were seven times more likely to have depression than those without such history according to the current study. In line with this finding, other studies [23, 38] reported that health workers who had a history of medication for mental health problems had a higher risk to exhibit anxiety, depression, and insomnia symptoms compared with those who had no psychiatric history.

In addition, HCWs with decrease in sleep hours were two times more likely to have depression, but depression is associated with decrease in sleep hours and the cross-sectional design of this study cannot answer what started first?

Finally, HCWs who have fear of COVID-19 infection for themselves or relatives were four times more likely to have depression more than HCWs who did not have this fear. Fear of death for HCWs or their relatives double the risk of depression among HCWs. These findings are supported by previous studies [21, 23, 28].

According to these findings, the mental health status of HCWs should be closely monitored by the Ministry of Health to facilitate the appropriate psychological care. They should be provided with appropriate safety measures, their workload should be managed and they should be compensated by the appropriate financial support, to reduce the mental health burden during such pandemic. This study had some limitations which include: first, cross-sectional design does not investigate causality; second, only 56% of the sample size was interviewed using SCID-I, which might limit the strength of using a structural interview instead of self-rating scales. However, this study has some strengths: first, it used structural interview based on SCID-I in addition to self-rating scales; second, it includes many health professions not only physicians.

## Conclusions

Three out of four HCWs who were caring for COVID-19 patients had major depression and two-thirds had severe symptoms. Young age, female gender, decreased sleep hours, past history of psychiatric illness, fear of infection or death due to COVID-19 were not only predictors of depression, but also of its severity. Screening for depression among HCWs, particularly for young females, and early treatment are recommended.

## Data Availability

Available upon request.

## Abbreviations

SCID: Structure Clinical Interview of DSM
HCWs: Healthcare workers
MDD: Major depression disorder
DASS: Depression Anxiety Stress Scale
WHO: World Health Organization
PPE: Personal protective equipment
